# Rare clinical image on steatocystoma multiplex in scrotum region

**DOI:** 10.11604/pamj.2024.47.202.43229

**Published:** 2024-04-22

**Authors:** Achal Maroti Gulghane, Vaishali Taksande

**Affiliations:** 1 Department of Obstetrics and Gynaecological Nursing, Smt. Radhikabai Meghe Memorial College of Nursing, Sawangi (Meghe), Wardha, India,

**Keywords:** Benign nodule, scrotal calcinosis, steatocystoma multiplex, calcium deposits, idiopathic scrotal calcinosis

## Image in medicine

A 41-year-old man came to the hospital with scrotal rashes that had been present for 15 years. The rashes have grown in size and number over time without causing any pain. There was no prior medical history that would have suggested a Sexually Transmitted Disease, trauma, or scrotal irritation. He does not take immunosuppressive medications, nor is he a known diabetic. There aren't any indicators of hypercalcemia. No history of steatocystoma multiplex in scrotum region disease or any other disease. On examination, tenderness was noted over the scrotum, and small nodules and rashes were present but no mass was felt. and the rest of the head-to-foot examination was normal. Routine blood parameters showed leucocytosis (15,000 WBC/mm^3^) and normal levels of urea (27mg%), creatinine (0.9 mg%), electrolytes (Na-140, K-3.4) and sugar (110mg%). physical assessment, the primary result came from a scrotal examination, which showed many nodular tumour’s affecting the scrotal surface, sparing the penis and other portions of the scrotum. The biggest nodule was around 8 mm by 6 mm in size. There were no sore spots or ulcers on the lesions. Multiple opacities were visible in the location of the lesions on the scrotal X-ray. The incisional biopsy's histology revealed calcium deposits encircled by pseudo capsules and histiocytic inflammation in the scrotum's dermis. aesthetic reasons, he asked to be removed. an excellent surgical result was achieved by wide local excision of the lesion followed by direct closure. The removed lesion's histology did not change. multiple opacities were visible in the location of the lesions on the scrotal.

**Figure 1:**
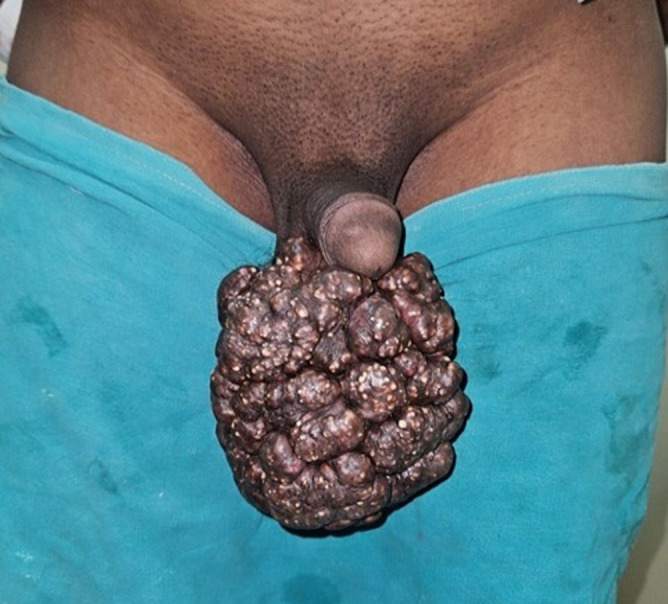
steatocystoma multiplex in scrotum

